# Andersen’s model on determining the factors associated with antenatal care services in Nepal: an evidence-based analysis of Nepal demographic and health survey 2016

**DOI:** 10.1186/s12884-020-02976-y

**Published:** 2020-05-19

**Authors:** Bidusha Neupane, Sujan Rijal, Srijana G.C., Til Bahadur Basnet

**Affiliations:** 1 Institute of Medicine, Tribhuwan University, Maharajgunj, Kathmandu, Nepal; 2 University of Oslo, Oslo, Norway; 3 BP Koirala Institute of Health Science, Dharan, Nepal

**Keywords:** Antenatal care, Equality, Health utilization, Andersen behavioral model

## Abstract

**Background:**

With the formulation of the National Safe Motherhood Policy in 1998, safe motherhood has forever been a priority program in Nepal. Under the safe motherhood program, every woman is provided with essential maternal health care services until now through the four-tire district health care system. There is a considerable increase in the utilization of antenatal care (ANC) by a skilled health provider from 2011 to 2016, 58 to 84%, respectively. However, inequality, exclusion, and under-utilization in health care services continue in many regions of Nepal. The present study aimed to explore the different types of socio-demographic factors associated with current ANC service utilization in Nepal.

**Methods:**

A cross-sectional study was conducted using the Nepal Demographic and Health Surveys data (DHS-7, 2016–2017). We estimated the latest pregnancy and live births in recent 5 years with the utilization of ANC services, and socio-economic differentials in these indicators under the framework of the Andersen behavioral model.

**Results:**

Two in three (69.8%) with last birth accessed at least four ANC visits. The rate of live birth was about 98.6% in the ANC4+ group, higher than that of 96.8% in the ANC4- group (χ2: 14.742, *P* <  0.001). In the multilevel logistic regression analysis, we found that women from province 2 (OR: 0.48; 95%CI: 0.32–0.74) and province 6 (OR: 0.46; 95%CI: 0.30–0.71) were significantly less likely to visit ANC 4 or more times. Age (OR: 0.95; 95%CI: 0.93–0.96) was also significantly associated with the frequency of ANC visits. Level of Women’s education and education of her partner were both significantly associated with the ANC visits: women (OR: 4.64; 95%CI: 3.05–7.05) and her partner (OR: 1.45; 95%CI: 1.01–2.06) having higher education were most likely to go for the recommended number of ANC visits. Moreover, women having exposure to multimedia were more likely to go for four or more ANC check-ups.

**Conclusions:**

The results highlight the need for governments and health care providers to develop special health promotion program with a focus on the vulnerable and disadvantaged and to use multi-media for maternal health literacy improvement flexibly, and maternal health system strengthening.

## Background

### Global context

An estimate from United Nations International Children’s Emergency Fund (UNICEF) in 2015 depicts that 800 women and 2700 newborns died every day due to the complication of pregnancy and childbirth. ANC and delivery by the skilled attendant are vital to prevent such deaths [1]. Compared to 59% in 1990, women in 2015 had more access to antenatal care services, where 71% of total women in the world can get access to antenatal care services [1]. Maternal deaths are being increasingly concentrated in sub-Saharan Africa and some South Asian nations, the nations that lack quality services [1]. Disparities exist all over the world in terms of access to service with poor, uneducated women coming from the lower wealth quintile having less access to much need ANC, and the disparity on access has not changed in the last 15 years. Hence, concrete and specific work addressing the issues related to inequality is needed to narrow this gap [1].

According to World Health Organization (WHO) Global Health Observatory, in 83 countries, 75% of women had at least four times ANC check-ups, while 86% of women had received ANC checkup at least once by the skilled professionals. In developed countries like the United States, France, and Canada, the figure ranges above 80 to 100%. While the statistics are quite different for developing nations, including African and some Asian countries, the number ranges from somewhere between 50 and 60% [2, 3]. A report published by WHO mentions that death due to maternal-related causes in a developing country is 33% higher than for women living in a developed country [2].

In 2015, Maternal Mortality Rate (MMR) in developing countries was 239 pregnant women per 100,000 live births, compared with 12 per 100,000 in developed countries [4]. While almost all pregnant women in high-income countries have had at least four prenatal visits, only 40% of pregnant women in low-income countries have received more than four prenatal care [5]. The MMR of the world decreased by 52.0% between 1980 and 2015, which indicated that the public health policy failed to meet the requirement of reducing the three-quarters of the Millennium Development Goals (MDGs). Currently, Sustainable Development Goals (SDGs) have a high ambition to reduce maternal mortality to less than 70/100,000 live births by 2030, with an annual decline rate of at least 7.5% [6]. Thus, there is a need for more focused intervention to save the lives of mothers and children. Evidence suggests that ANC checkup in the past has substantially contributed a lot to address the issues; an improved ANC checkup could potentially mean a better maternal and child health outcome [5–7]. For instance, at least four ANC visits, which include blood pressure measurement, blood, and urine tests and advice on pregnancy complications and advice on where to go if such complications, has shown to decrease the risk of neonatal mortality [8, 9]. In an Indian study, compared to women who had neonatal deaths, women who had given live birth had received better quality recommended number of ANC [10].

### Nepal’s context

With the formulation of the National Safe Motherhood Policy in 1998, safe motherhood has forever been a priority program in Nepal. Under the safe motherhood program, every woman is provided with essential maternal health care services until now through the four-tire district health care system [11]. Due to the intense policy action which attracted a lot of programs and fund in the field of maternal and child health care, we have managed to increase the coverage of maternal health services over the years [12–14]. With such an implementation of effective policy, Nepal has been one of the few low- and middle-income countries that achieved several MDGs well before 2015 with an increase in the utilization of ANC by skilled health providers from 2011 to 2016, 58 to 84% respectively [15]. However, inequality, exclusion, and under-utilization in health care services continue in regions of Nepal [16]. Almost 15% of Nepalese women reported no ANC visits, and only half (50%) reported four or more ANC visits, The recommended number of visits is not always met [17]. A study conducted in Nepal (2013) revealed that educated women of younger age whose husbands were also educated, living in urban areas, from non-farming occupations and falling in higher wealth quintiles were more likely to attend four or more ANC and receive higher quality ANC [8]. Very few studies focus on the quality of ANC in low-income countries like Nepal, and most of them have focused only on individual factors [18, 19]. The present study was conducted to explore different types of contextual and individual factors associated with current ANC service utilization in Nepal, to provide evidence-based information to address the problems more precisely on improving the equity and coverage of essential maternal health service utilization.

### Conceptual framework

**Fig. 1:**
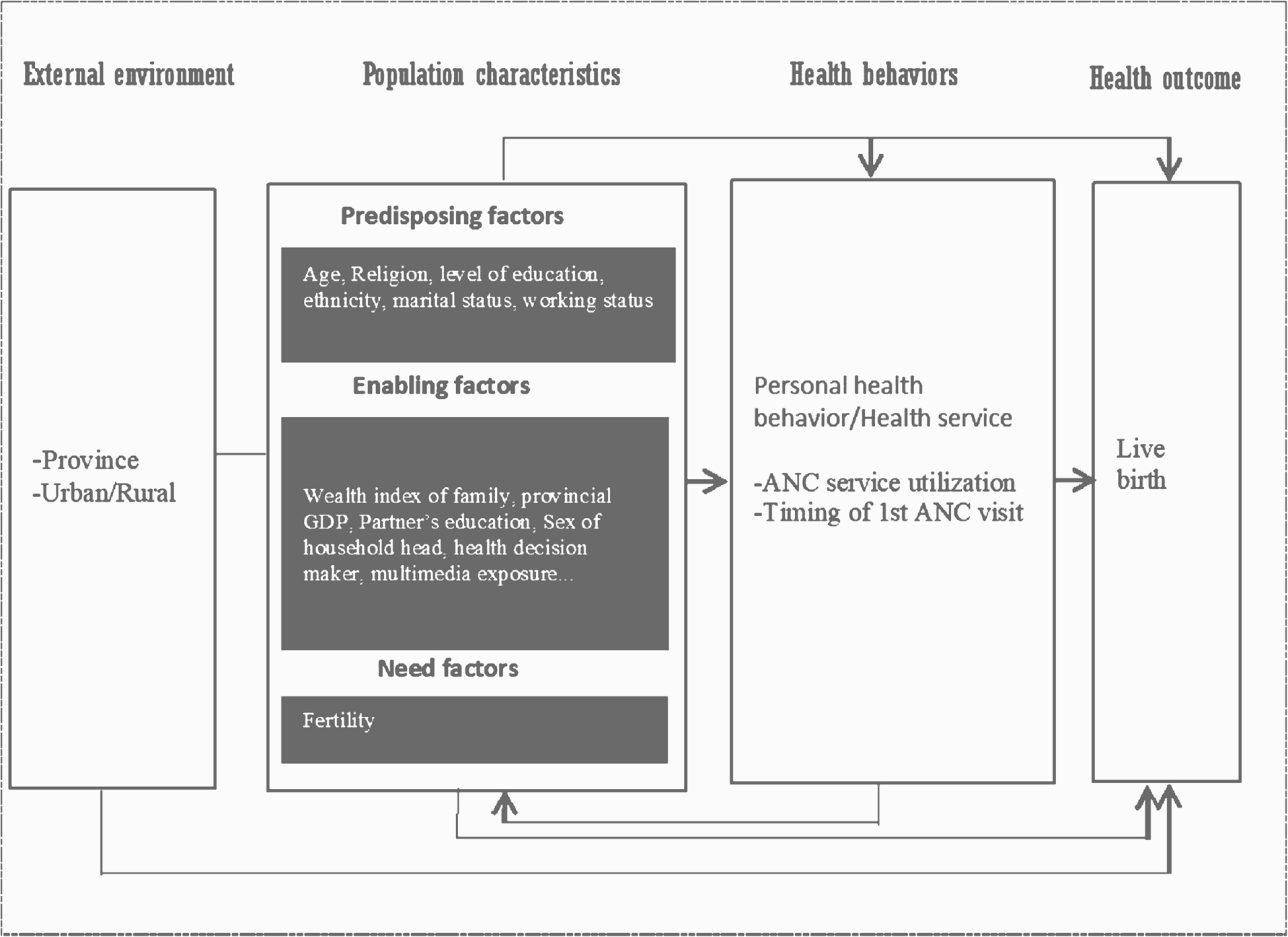
Conceptual Framework of Anderson behavioral model. GDP: Gross domestic product; ANC: Antenatal care

The framework of the Andersen behavioral model was adopted for classifying the factors associated with ANC visits. This multilevel model incorporated individual and contextual determinants of health services use and was developed by Ronald M. Andersen in 1968 and advanced in 1990 (the fourth version) [20, 21]. It had different layers, including the external environment, population characteristics, and health behavior, and health outcome (Fig. 1).

## Methods

### Data source

We used data from the 2016 Nepal Demographic and Health Survey (NDHS), which is a national level household survey. Under the Ministry of Health, the survey began in June 2016 and lasted until January 2017. A total of 11,473 households were selected for the sample, where, 11,203 were occupied. From those households, 11,040 were successfully interviewed. Among the interviewed households, 13,089 women age 15–49 were identified for individual interviews; interviews were completed with 12,862 women, with a response rate of 98%. We restricted our analyses to individual women age 15–49 of reproductive age who given birth 5 years before NDHS 2016. Women of age group 15–49 selected for the study either were permanent residents of the household selected for an interview or an eligible visitor who had stayed there a night before the survey [15].

The data collection for the NDHS, 2016, was ethically approved by the Nepal Health Research Council (NHRC), which is a national level government organization leading in research activities in Nepal. Similarly, ethical clearance was also obtained from ICF Macro Institutional Review Board, Maryland, USA. Data were collected after an online application was submitted to the demographic health survey (DHS) program explaining the purpose of the study, intended use, and people who would have access over the data [15]. After the review of the online application, permission had been obtained from the monitoring and evaluation body of DHS globally, MEASURE DHS, to use the data set for this study. The NDHS, 2016 data are publicly available at the USAID DHS program (at http://dhsprogram.com/data) in different formats.

### Outcome variable

Our outcome variable was the attendance of at least four or more times ANC (ANC 4+) which is the recommended number of ANC visits by WHO.

### Study variables

Altogether 15 variables relevant to the study were selected and divided into contextual factors and individual factors [22, 23]. Nepal has recently adopted the federal system of government, whereby the formal names of the provinces are yet to be decided, hence, they are still known by numbers through 1 to 7. Province of residence, place of residence (urban/rural), age of the women, ethnicity (Brahmin/Chettri, Janjati, Dalit, Muslim and others), religion (Hindu, non-Hindu), women’s education (no education, primary, secondary and higher), partner’s education (no education, primary, secondary and higher), respondent’s occupation (didn’t work, skilled worker, unskilled worker, and agriculture), household wealth index (poor, middle class, rich), provincial GDP, sex of household head (male, female), health care decision-maker (women herself, herself along with someone else, solely others), exposure to the newspaper (not at all, less than once a week, at least once a week), exposure to the radio (not at all, less than once a week, at least once a week) and exposure to television (not at all, less than once a week, at least once a week).

### Statistical analyses

A total of 4006 ever-married women between 15 and 49 years were eligible to be included in our study. We estimated the latest pregnancy and live birth in recent 5 years with the utilization of ANC service and the socio-economic differentials in these indicators by age, work, residence, religion, wealth, media exposure were analyzed using a multilevel logistic regression model.

The variables were divided as per the need for predisposing and enabling factors. After that, descriptive statistics were performed to find the frequency of various independent variables. A Chi-square test was executed to find the relation between the dependent variables (ANC4+) and the outcome variable (live birth). Furthermore, the variables were divided into contextual factors and individual factors for further analyses. Province, provincial GDP, household wealth index, religion, and ethnicity were considered as the contextual factors while the rest were the individual factors. Since GDP was not available from NDHS, the GDP of 2011 was taken from a data source that contains data related to Nepal [24]. The multilevel nested structure of analysis comprised 4006 individuals (level 1) grouped into 380 primary sampling units (PSUs), which were wards in rural areas and enumerator areas in the urban area (level 2). Again, the PSUs were nested into the place of residence (urban and rural area) (level 3). Multilevel logistic regression was performed to test the association of contextual and individual independent variables with the number of 4^+^ANC visits in Nepalese pregnant women. Variables associated with 4^+^ANC at a significant level *p* < .05 were considered for the multivariable analysis. A three-level random intercept and fixed-slopes model structure with individuals nested within PSUs and PSUs within urban-rural cities were fitted to estimate the odds ratios (OR) and 95% CIs, indicating the likelihood of having a higher mean of 4^+^ANC visit. The overdispersion of the data was handled by using a three-level random intercept model.

Stepwise forward selection of variables in subsequent models was conducted to obtain a parsimonious final model for ANC visit, according to the theoretical framework (Fig. 1). The first and second models consisted of contextual predisposing and enabling factors, and the second and third models comprised of individual predisposing and enabling variables, respectively. Variables that remained statistically significant at 5% (*P* ≤ .05) were retained in the analysis for adjustment in the next model. Thus, the final models included all significant contextual and individual predisposing and enabling factors. The statistical analyses were executed using R version 3.6.3 with the “lme4” package.

## Results

### Respondent’s socioeconomic and demographic characteristics

Table 1 shows the participant’s socioeconomic and demographic characteristics. Seventy-five percent of total pregnant women living in urban parts of Nepal went for four or more ANC visits compared to 62% from rural regions. Similarly, for the province of residence, the percentage distribution of 4 or more times ANC visit was almost the same for provinces 1,3,4,5 and 7. The percentage was relatively low in province 2 and 6. There was a significant association of ANC 4^+^ with the province of residence and if the women came from a rural or urban area (*p* <  0.001).

**Table 1:** Respondent’s characteristics divided into environmental, predisposing and enabling factors

Variable	Total	No ANC (%)	1–3 times ANC (%)	4+ ANC (%)	***p***-value
**Environmental Factor**					
**Province**					
Province 1	575	26 (4.5)	103 (17.9)	446 (77.6)	< 0.001
Province 2	759	37 (4.90)	302 (39.8)	420 (55.3)	
Province 3	434	28 (6.5)	70 (16.1)	336 (77.4)	
Province 4	436	29 (6.7)	74 (17)	333 (76.4)	
Province 5	651	32 (4.90	137 (21)	482 (74)	
Province 6	602	74 (12.3)	170 (28.2)	358 (59.5)	
Province 7	549	26 (4.7)	103 (18.8)	420 (76.5)	
**Residence**					
Urban	2338	112 (4.8)	465 (19.9)	1761 (75.3)	< 0.001
Rural	1668	140 (8.4)	494 (29.6)	1034 (62)	
**Predisposing Factor**					
**Age years**					
15–19	344	10 (2.9)	79 (23)	255 (74.1)	< 0.001
20–29	2657	130 (4.9)	624 (23.5)	1903 (71.6)	
30–49	1005	112 (11.1)	256 (25.5)	637 (63.4)	
**Ethnicity**					
Brahmin/Chettri	1361	76 (5.6)	241 (17.7)	1044 (76.7)	< 0.001
Janjati	1266	92 (7.3)	265 (20.9)	909 (71.8)	
Dalit	584	42 (7.2)	166 (28.4)	376 (64.4)	
Muslim	229	7 (3.1)	101 (44.1)	121 (52.8)	
Others	566	35 (6.2)	186 (32.9)	345 (61)	
**Religion**					
Hindu	3487	225 (6.5)	796 (22.8)	2466 (70.7)	< 0.001
Non-Hindu	519	27 (5.25)	163 (31.4)	329 (63.4)	
**Level of education**					
No education	1231	153 (12.4)	452 (36.7)	626 (50.90)	< 0.001
Primary	763	54 (7.1)	215 (28.2)	494 (64.7)	
Secondary	1396	40 (2.9)	249 (17.8)	1107 (79.3)	
Higher	616	5 (0.8)	43 (7)	568 (92.2)	
**Respondent’s Occupation**					
Didn’t work	1441	95 (6.6)	347 (24.1)	999 (69.3)	0.217
Skilled worker	466	36 (7.7)	94 (20.2)	336 (72.1)	
Unskilled worker	114	9 (7.9)	32 (28.1)	73 (64)	
Agriculture	1985	112 (5.6)	486 (24.5)	1387 (69.9)	
**Enabling Factor**					
**Wealth Index of family**					
Poor	1879	200 (10.6)	520 (27.7)	1159 (61.7)	< 0.001
Middle	822	22 (2.7)	225 (27.4)	575 (70)	
Rich	1305	30 (2.3)	214 (16.4)	1061 (18.3)	
**Provincial GDP per capita ($)** (USD)					
< 718 (national average) level)	2561	169 (6.6)	712 (27.8)	1680 (65.5)	< 0.001
718–1000	1011	55 (5.4)	177 (17.5)	779 (77.1)	
> 1000	434	28 (6.5)	70 (16.1)	336 (77.4)	
**Partner’s Education Status** (*n* = 3130)					
No education	504	65 (12.9)	196 (38.9)	243 (48.2)	< 0.001
Primary	840	81 (9.6)	252 (30)	507 (60.4)	
Secondary	1867	80 (40.3)	397 (21.3)	1390 (74.5)	
Higher	2626	17 (2.3)	105 (13.9)	632 (83.8)	
**Sex of household head**					
Male	2746	173 (6.3)	680 (24.8)	1893 (68.9)	0.187
Female	1181	79 (6.3)	279 (22.1)	902 (71.6)	
**Healthcare Decision maker**					
Herself	848	59 (7)	166 (19.6)	623 (73.5)	0.003
Women and someone else	1149	58 (5)	274 (23.8)	817 (71.1)	
Others	1973	126 (6.4)	511 (25.9)	1336 (67.7)	
**Exposure to Newspaper**					
Not at all	3033	237 (7.8)	868 (28.6)	1928 (63.6)	< 0.001
Less than once a week	791	14 (1.8)	81 (10.2)	696 (88)	
At least once a week	182	1 (0.5)	10 (5.5)	171 (94)	
**Exposure to Radio**					
Not at all	1696	135 (8)	540 (31.8)	1021 (60.2)	< 0.001
Less than once a week	1271	67 (5.3)	242 (19)	962 (75.7)	
At least once a week	1039	50 (4.8)	177 (17)	812 (78.2)	
**Exposure to Television**					
Not at all	1519	168 (11.1)	501 (33)	850 (56)	< 0.001
Less than once a week	893	43 (4.8)	217 (24.3)	633 (70.9)	
At least once a week	1594	41 (2.6)	241 (15.1)	1312 (82.3)	

The percentage of four or more ANC visits was highest (74.1%) among the 15–19 years age group and lowest among women beyond 35 years of age at 54.1%. The women from the Muslim community were the lowest contributor; at 53.1% four or more ANC visits. Here, age, ethnicity, level of education, and religion were significantly associated with 4^+^ ANC use (*p* <  0.001).

Only 65.6% of the total pregnant women who resided in provinces whose provincial GDP per capita was below the national average (i.e., $718) went for four or more ANC visits. Likewise, 77.2% of women with educated partners went for four or more ANC visits. A higher percentage of women, i.e., 94% of pregnant women with exposure to the newspaper at least once a week, went for four or more ANC visits compared to only 63.6% of those who did not have access to the newspaper at all. Household wealth index, provincial GDP, partner’s education, participation in healthcare decision making, exposure to radio, TV, and newspaper were all significantly associated with ANC 4+ visit (*p* <  0.001).

**Fig. 2:**
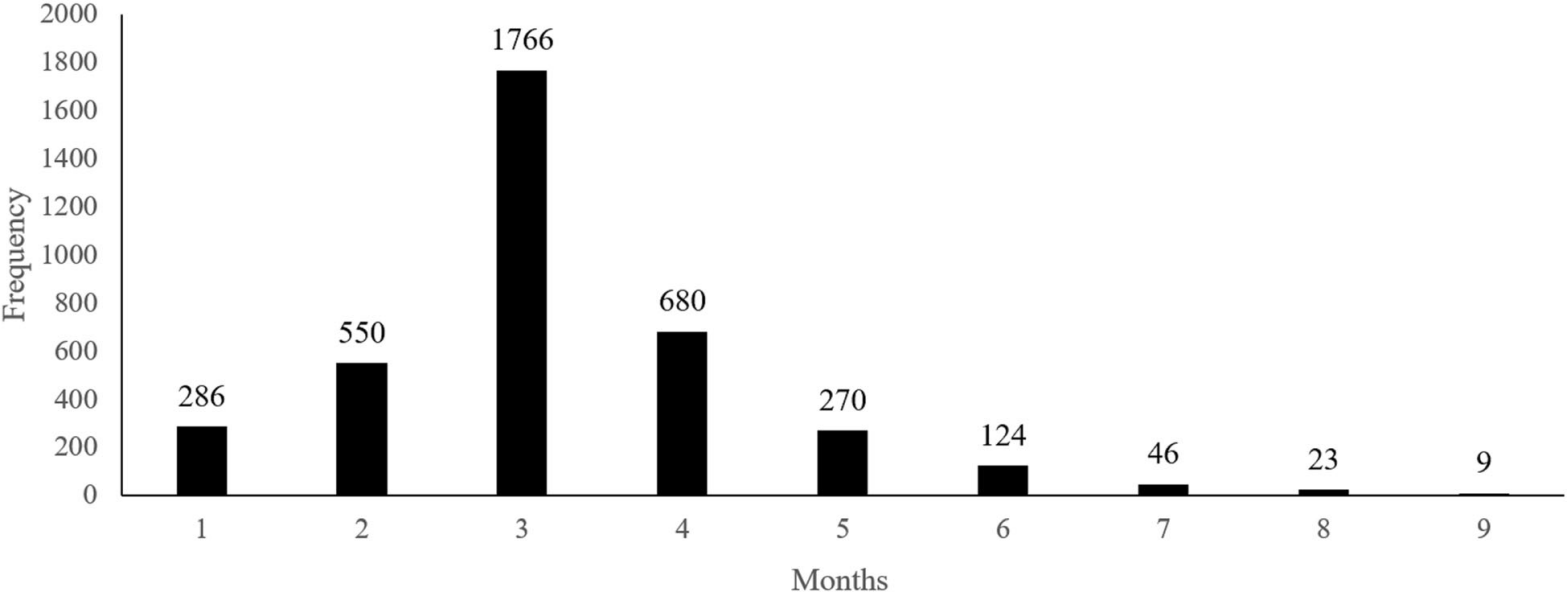
Timing of 1st antenatal check among times of ANC visits ≥1 group. ANC: Antenatal care; *N* = 3754

As many as 3754 women, i.e., 94% of total women who were pregnant 5 years before the survey, have visited for an antenatal check-up at least once during their pregnancy. Among the total women, 69.8% of total women have visited for ANC check-up four or more times as per the recommendation, while 6.3% of women had no ANC visits (Fig. 2).

The birth outcome being live was higher in the women who had visited for ANC check-up four or more times than that of women who went for ANC check-up less than four-times (*χ*^*2*^: 14.742, *p* <  0.001) (Table 2)**.** For the women, who had visited for ANC check-up more than once or at least, most of the women had their first visit during the 3rd month of the pregnancy, followed by the 4th month and then 2nd month. Few women also had the 1st visit on the 7th, 8th, or 9th month (Fig. 2).

**Table 2:** Association of times of ANC visits with live birth

Times of ANC visits	Total (***N*** = 4006)	Live birth (%)	χ^**2**^	***p***-value
0–3	1211	1172 (96.8)		
4+	2795	2756 (98.6)	14.742	0.001

### Multilevel logistic regression between ANC 4^+^ and contextual and individual factors

Table 3 presents the result of the multilevel logistic regression model for the frequency of ANC visits. Model 1 shows the effect of contextual factor (predisposing) on the propensity of completing four or more ANC visits, which predicted women from province 2, 6 and 7, and women coming from other ethnic backgrounds besides Brahmin/Chettri were significantly less likely to visit ANC 4 or more times. In model 2, the effect of contextual factors (predisposing and enabling) on the frequency of ANC visits where women from province 2 and 6, and women from every ethnicity. As compared to Brahmin/Chettri, all other ethnic pregnant women had a significantly low probability of going for at least a minimum recommended number of ANC, while women from the middle and wealthy class were significantly more likely to go for four or more ANC check-ups.

**Table 3:** Multilevel logistic regression of factors associated with ANC 4+

Variables	Model 1 OR (95%CI)	Model 2 OR (95%CI)	Model 3 OR (95%CI)	Model 4 OR (95%CI)
**Contextual variables**				
**Predisposing variables**				
**Provinces**				
Province 1 *(ref)*				
Province 2	0.35 (0.22–0.57)^a^	0.35 (0.22–0.54)^a^	0.44 (0.28–0.67)^a^	0.48 (0.32–0.74)^b^
Province 3	1.11 (0.68–1.8)	1 (0.63–1.56)	1.04 (0.66–1.64)	1 (0.63–1.57)
Province 4	0.97 (0.59–1.58)	0.94 (91.06–0.84)	0.83 (0.53–1.3)	0.78 (0.5–1.22)
Province 5	0.89 (0.56–1.4)	0.83 (0.54–1.27)	0.9 (0.59–1.37)	0.93 (0.61–1.41)
Province 6	0.35 (0.22–0.55)^a^	0.44 (0.28–0.69)^a^	0.46 (0.3–0.71)^a^	0.46 (0.3–0.71)^c^
Province 7	0.61 (0.38–0.98)^c^	0.73 (0.47–1.14)	0.92 (0.59–1.42)	0.94 (0.61–1.46)
**Ethnicity**				
Brahmin/Chettri *(ref)*				
Janjati	0.48 (0.37–0.61)^a^	0.52 (0.41–0.67)^a^	0.66 (0.51–0.85)^b^	0.67 (0.52–0.87)b
Dalit	0.48 (0.36–0.63)^a^	0.57 (0.44–0.76)^a^	0.82 (0.62–1.09)	0.88 (0.66–1.18)
Muslim	0.34 (0.19–0.61)^a^	0.28 (0.17–0.44)^a^	0.49 (0.31–0.79)^b^	0.57 (0.36–0.91)^c^
Others	0.53 (0.37–0.75)^a^	0.49 (0.34–0.69)^a^	0.67 (0.47–0.97)^c^	0.74 (0.51–1.07)
**Religion**				
Hindu *(ref)*				
Non--Hindu	0.92 (0.65–1.29)			
**Enabling factors**				
**Wealth Index of family**				
Poor *(ref)*				
Middle		1.88 (1.5–2.36)^a^	1.6 (1.27–2.03)^a^	1.38 (1.08–1.76)
Rich		2.87 (2.29–3.6)^a^	1.97 (1.56–2.49)^a^	1.53 (1.19–1.99)
**Individual factors**				
**Predisposing factors**				
**Age**			0.95 (0.94–0.96)^a^	0.95 (0.93–0.96)^a^
**Level of education**				
No education *(ref)*				
Primary school			1.64 (1.32–2.03)^a^	1.38 (1.1–1.72)^b^
Secondary school			2.78 (2.26–3.43)^a^	1.87 (1.47–2.36)^a^
Higher school			7.7 (5.37–11.04)^a^	4.64 (3.05–7.05)^a^
**Enabling factors**				
**Level of partner’s education**				
No education *(ref)*				
Primary school				1.24 (0.95–1.61)
Secondary school				1.51 (1.17–1.95)^b^
Higher school				1.45 (1.01–2.06)^c^
**Healthcare Decision maker**				
Herself *(ref)*				
Women and someone else				0.94 (0.74–1.2)
Others				1.06 (0.86–1.32)
**Exposure to Media**				
Less than once a week *(ref)*				
At least once a week				1.86 (0.94–3.66)^b^
**Variance at individual: (PSU: residence (urban/rural)**: **PSU)**	0.0006	0.0002	< 0.00001	< 0.00001
**Variance at PSU: residence (urban/rural)**	0.75	0.62	0.53	0.51
**Variance at residence (urban/rural)**	0.123	0.05	0.021	0.02
**ICC**	0.21	0.17	0.143	0.13

Model 1: mutually adjusted for contextual predisposing variables

Model 2: mutually adjusted for contextual predisposing and enabling variables

Model 3: mutually adjusted for contextual predisposing and enabling variables, and individual predisposing variables

Model 4: mutually adjusted for contextual predisposing and enabling variables, and individual predisposing and enabling variables

*Ref*: Reference, *OR* Odds ratio, *CI* Confidence interval, *PSU* Primary sampling unit, *ICC* Intra class correlation coefficient

^c^*P* ≤ 0.05; ^b^*P* ≤ 0.01; ^a^*P* ≤ 0.001

Individual-level predisposing variables are incorporated in model 3, and the result showed province, ethnicity, level of education, and age were significantly associated with the frequency of ANC visit. Women who had primary, secondary, or higher education had a significantly higher probability of going for ANC check-ups four or more times. Finally, both contextual (predisposing and enabling) and individual factors (predisposing and enabling) were incorporated in model 4, where province, ethnicity, age, level of education (women’s), partner’s education and exposure to the newspaper, radio, and TV were significantly associated with the frequency of ANC visit. Pregnant women’s partners who had received secondary or higher education were significantly more likely to go for the recommended number or more ANC check-ups**.**

Regarding variances for each of the random effects in the models, model 1 shows that most of the variation was observed at PSU within residence type, which was, even though decreasing across the subsequent model with the addition of individual factors. Decreasing variance at residence type also was observed through model 1 to model 4. Similarly, intra class correlation coefficient was reported to decreasing across the model 1 to model 4.

## Discussion

The study differs from other similar studies in terms of analysis; we have carried out multilevel logistic regression to analyze the relative contribution of contextual factors and individual factors [8, 12]. The twofold analyses could contribute to defining an appropriate level of intervention and designing effective policies.

Our study, like other studies, shows that birth outcomes to be significantly better in women who visit ANC as per the recommendation [25–30]. Increased survival is one of the essential benefits of ANC to babies [31, 32]. Similarly, through this study, we found out the place of residence, age, religion, wealth index of the family, educational level, partner’s education level, and exposure to multimedia was significantly associated with a higher frequency of ANC visits in both contextual and individual models.

Recent studies conducted in Ghana showed that women in urban areas have a higher socioeconomic status, which was positively associated with higher use of ANC services [33]. This phenomenon can be explained by the urban effect where half of the urban impact was described by wealth and education alone [33, 34]. In a study conducted in Mumbai, India also found out the customs as per ethnicity or religion to influence the uptake of maternal health care services, which were similar to our study [35–37].

Women who came from a wealthy background were more likely to go and get an ANC checkup for four or more times, which is similar to another study conducted in Nepal [8]. There have scarce studies to show the relationship between the province and service utilization. Other provinces of Nepal comprise of more developed districts compared to provinces 2 and 6. Most districts in provinces 2 and 6 are relatively economically backward and geographically remote; longer the distance or difficulty the geography, women are less likely to go and use the services. Similarly, province 6 includes all of the remote western parts of Nepal, causing the rural-urban effect of lower service utilization in that part rural and economically backward provinces. Women in those parts gave birth in an animal shed due to the belief that deity would be angry if they would give birth at home [38], which could be the reason for lower education and economic status. Economic status directly affects the availability of services and education status of the women in the provinces and also affects the ability of a person to utilize the available services [39, 40].

The study also revealed that the strong association between the frequency of use of multimedia and ANC service utilization. Even if the women used any form of multimedia even less than once a week, they were at better odds of visiting for ANC four times or more. Hence, we concluded that exposure to multimedia could affect the service utilization of women in terms of maternal health. The exposure to multimedia and the utilization of maternal health services is understudied in Nepal. However, a study showed a 6-fold increase in the use of ANC with exposure to multimedia like television and radio [41]. Similar results have been observed in the studies conducted in countries like Bangladesh, India, and Uganda multimedia having a positive impact on ANC service utilization [18, 42–44].

Hence, better antenatal care results in a better birth outcome, which directly or indirectly contributes to maternal as well as child health. Various studies have explored the factors associated with the use of maternal services and have rooted common factors such as age, religion, economic status, etc. Our research has also observed the use of multimedia in relation to ANC visit, and we strongly suggest utilizing factors such as multimedia use to address the gap that lies in the utilization of ANC as per recommendation. A further study focusing on the use and content of media could lead us to a new direction on efforts to address the gaps associated with this.

The present study is a cross-sectional study where we cannot establish a causal relationship. The study is a retrospective study; hence, there are chances of recall bias whereby we have only analyzed the data regarding the most recent pregnancy within the past 5 years to minimize the recall bias. Similarly, this study did not take into account other relevant factors associated with the quality of antenatal care, such as the timing of visits and care practice during the visits.

However, we used NDHS data where the samples were taken across the nation so the findings can be generalized to the entire country. Also, the methodology used in NDHS is precise with stratified multilevel sampling and the use of the standard questionnaire [15]. Similarly, training was provided to enumerators before the data collection. All the ethical issues have been addressed before the collection of the data by DHS, and approval from institutional review board was taken [15].

## Conclusions

This study shows a strong association between both contextual and individual determinants and frequency of ANC visits. Contextual factors such as province, household wealth index, ethnicity, and individual level predisposing factors such as age along with enabling factors (partner’s education level, exposure to multimedia) contributed to the recommended number of ANC visits. Thus, we conclude that ANC attendance is vital for live birth, and intervention target at various levels is recommended.

Effective National Safe Motherhood Policy meets SDGs’ target by addressing healthy reproductive needs. To make National Safe Motherhood policy effective, the factors associated with 4+ ANC visit needs to be addressed appropriately with developing special health promotion program with a focus on the vulnerable and disadvantaged. Moreover, the flexible use of multimedia should be encouraged to improve maternal health literacy.

## Data Availability

The datasets generated and analyzed during the current study are available in the demographic and health survey (DHS) programme repository, www.dhsprogram.com/data/dataset_admin/login_main.cfm.

## Abbreviations

ANC: Antenatal Care
CI: Confidence Interval
DHS: Demographic Health Survey
GDP: Gross Development Product
MMR: Maternal Mortality Rate
MDGs: Millennium Development Goals
NDHS: Nepal Demographic and Health Survey
OR: Odds Ratio
PSUs: Primary Sampling Units
SDG: Sustainable Development Goal
USAID: United States Aid for International Development
WHO: World Health Organization
